# Treatment of a Case of Blood Poison

**Published:** 1892-02

**Authors:** Samuel H. Green

**Affiliations:** Oakland, Ga.


### TREATMENT OF A CASE OF BLOOD POISON.

BY SAMUEL H. GREEN, M. D., OAKLAND, GA.

I received into the penitentiary on June 30th, 1891, M. T. W., white, who, at the time, was affected with a chronic case of blood-poisoning, of two years standing. Patient had not passed his water in two weeks, nor had an action in six days.

The same day I removed two large ulcers from his right leg, and the following day, another from his right foot. I then gave him a large dose of saccharated calomel, which produced a free evacuation. Also, the following prescription:

℞ Pot. Acetat. dr. i.
Tr. Buchu, dr. ss.
Spts. Eth. Nit. dr. ss.
Aquæ q. s. dr. ii.

M. Sig. Teaspoonful three times daily.

In twelve hours he passed his water with perfect ease. I dressed his foot and leg with pulv. iodoform, sweet oil and gum camphor, then applying iodoform gauze each day, after being thoroughly cleansed with carbolized water, and a solution of bi-chloride of mercury. He at once began to improve, his appetite becoming better, and the ulcers healing up nicely. The seventh day after being received, the ulcers were entirely healed, and his bowels and kidneys in perfect condition. On the 7th day of July, I put him on this preparation;

R. Pot. Iodidi, dr. ss.
Tr. Gentian, dr. ss.
Aqua, qs. dr. ii.

M. Sig. Teaspoonful three times daily.

He improved under this treatment very fast. On the 15th day of July, I removed a portion of the tibia. The following day I gave him for incessant vomiting:

R Tr. Opii Camp. dr. iss.
Bismuth, Sub. Nit. dr. ss.
Tr. Ferri Mur. dr. ss.
Aquæ qs. dr. iii.

M. Sig. Teaspoonful three times a day.

On the 2nd of August, an abscess bursted in his left side, from which I got one pint of pus. Following, he had three convulsions, and began to sink. I injected into the rectum warm water, laudanum and starch, and applied hot poultices to his bowels, whereupon he began to resuscitate. I then gave large doses of salts. By way of diet, I ordered beef tea, toasted light bread, sweet milk and butter. He then began to improve under the treatment, and on the 30th day of November, 1891, I pronounced him a well man. However, the ankle and knee joints of both legs are ankylosed.

